# Modulation of Neurally Mediated Vasodepression and Bradycardia by Electroacupuncture through Opioids in Nucleus Tractus Solitarius

**DOI:** 10.1038/s41598-018-19672-9

**Published:** 2018-01-30

**Authors:** Stephanie C. Tjen-A-Looi, Liang-Wu Fu, Zhi-Ling Guo, John C. Longhurst

**Affiliations:** Susan Samueli Integrative Health Institute, School of Medicine, University of California, Irvine, CA 92697–4075 USA

## Abstract

Stimulation of vagal afferent endings with intravenous phenylbiguanide (PBG) causes both bradycardia and vasodepression, simulating neurally mediated syncope. Activation of µ-opioid receptors in the nucleus tractus solitarius (NTS) increases blood pressure. Electroacupuncture (EA) stimulation of somatosensory nerves underneath acupoints P5–6, ST36–37, LI6–7 or G37–39 selectively but differentially modulates sympathoexcitatory responses. We therefore hypothesized that EA-stimulation at P5–6 or ST36–37, but not LI6–7 or G37–39 acupoints, inhibits the bradycardia and vasodepression through a µ-opioid receptor mechanism in the NTS. We observed that stimulation at acupoints P5–6 and ST36–37 overlying the deep somatosensory nerves and LI6–7 and G37–39 overlying cutaneous nerves differentially evoked NTS neural activity in anesthetized and ventilated animals. Thirty-min of EA-stimulation at P5–6 or ST36–37 reduced the depressor and bradycardia responses to PBG while EA at LI6–7 or G37–39 did not. Congruent with the hemodynamic responses, EA at P5–6 and ST36–37, but not at LI6–7 and G37–39, reduced vagally evoked activity of cardiovascular NTS cells. Finally, opioid receptor blockade in the NTS with naloxone or a specific μ-receptor antagonist reversed P5–6 EA-inhibition of the depressor, bradycardia and vagally evoked NTS activity. These data suggest that point specific EA stimulation inhibits PBG-induced vasodepression and bradycardia responses through a μ-opioid mechanism in the NTS.

## Introduction

Vasovagal syncope, in particular, neurally mediated syncope^1^, that can be modulated by electroacupuncture (EA) is incompletely understood . The pathophysiology of neurally mediated syncope includes activation of cardiopulmonary afferent neuronal pathways and medullary cardiovascular centers^2^. Central processing in these centers and subsequent efferent neural activation result in severe bradycardia and vasodepression^3,4^. Pharmacological therapy such as β-adrenergic blockers and serotonin reuptake inhibitors^5,6^ are used to treat patients with recurrent syncope. Treatment and evaluation of syncope cost over $2.4 billion in the USA in year 2000^7^. Furthermore, treatment is often unsatisfactory because of medication side effects or recurrent symptoms^8^. In search of other possible therapies, we have shown that stimulation of somatic sensory nerves by acupuncture has the potential to reduce the decreased heart rate (HR) and blood pressure (BP)^1,9–11^. We have shown that EA modulates bradycardia through medullary processing, but the neural mechanisms underlying these actions are unclear^1,4^.

Electroacupuncture at the P5–6 acupoints applied for 30 min using low frequency and low voltage reduces reflex induced hypertension, hypotension or vasodilation and myocardial ischemia^4,9,12–16^. Stimulation at these acupoints alters afferent discharge of the median nerves and subsequent neural activity in cardiovascular regions of the central nervous system (CNS) to influence the autonomic outflow. These actions have the potential to reverse adverse hemodynamic responses^17,18^. In this regard, reflex hypertension mediated through sympathoexcitatory pathways in the CNS is reduced by acupuncture at P5–6^19–21^ through the actions of several neurotransmitter systems, including endocannabinoids, opioids, serotonin and γ-aminobutyric acid (GABA), among others, in hypothalamic, midbrain and medullary cardiovascular regions^16,20–23^.

Reflex vasodepression and bradycardia evoked by activation of cervical and abdominal vagal afferents are mediated by parasympathetic pathways^4,24^. More specifically, decreases of BP and HR are induced by stimulation of pulmonary vagal C-fibers with adenosine, capsaicin, hypercapnia, hemorrhage or phenylbiguanide (PBG)^25–27^. Similar to its actions on reflex sympathoexcitatory responses, EA at P5–6 acupoints also reduces parasympathoexcitatory reflex responses, i.e. vasodepression and bradycardia^4,9^. Thus, EA at P5–6 acupoints on the wrist overlying the median nerves modulates both excitatory and inhibitory cardiovascular reflex responses.

We have demonstrated that acupuncture stimulation of the deep somatic nerves, including the median nerves positioned under P5–6 acupoints, inhibits pressor and depressor responses while stimulation of points overlying cutaneous nerves do not influence the sympathoexcitatory and parasympathoinhibitory responses^4,16^. For instance, EA stimulation of the forelimbs at LI6–7 overlying the superficial radial nerve, in contrast to its actions at P5–6, does not normalize pressor responses^14^. Similarly, EA at P5–6, but not LI6–7, reduces gastric-distention induced hypotension in rats subjected to hypercapnia^9^. However, it is unknown if the Bezold Jarisch cardiopulmonary reflex responses, important in the onset of neurally mediated syncope^2,3^, display similar point specific responses to EA.

Medullary nuclei, including the rostral and caudal ventrolateral medulla (rVLM and cVLM), medullary nucleus ambiguus (NAmb) and nucleus tractus solitarius (NTS) process cardiopulmonary vagal afferent input that ultimately lead to inhibitory hemodynamic responses^1,4,28,29^. The Bezold Jarisch reflex responses induced with i.v. PBG activate cardiopulmonary serotonin receptors in vagal afferents^3^. While we have shown that the long-lasting EA effect reduces PBG-induced bradycardia and depressor responses for over an hour^1,4^, the central mechanisms are unclear. We do know that GABA in the NTS participates in EA inhibition of reflex associated bradycardia but not vasodepression^1^. Furthermore, activation of µ-opioid receptors within the NTS increases BP^30^. However, nothing is known about the mechanisms underlying EA-inhibition of vasodepression during the Bezold Jarisch response. Additionally, there is no information available about point specific actions of EA on reflex cardioinhibitory responses mediated through central parasympathetic nuclei in the brain stem. In the present study we hypothesized that, similar to EA’s actions on sympathetic outflow during sympathoexcitatory reflex events, i.e., EA-stimulation at P5–6 or ST36–37 but not LI6–7 or G37–39 acupoints, inhibits the bradycardia and vasodepression and that µ-opioid receptors in the NTS mediate EA-inhibition of PBG-evoked responses. This work has been published in preliminary form^31^.

## Results

### NTS opioid system in EA-inhibition of cardiopulmonary reflex responses

**Figure 1:**
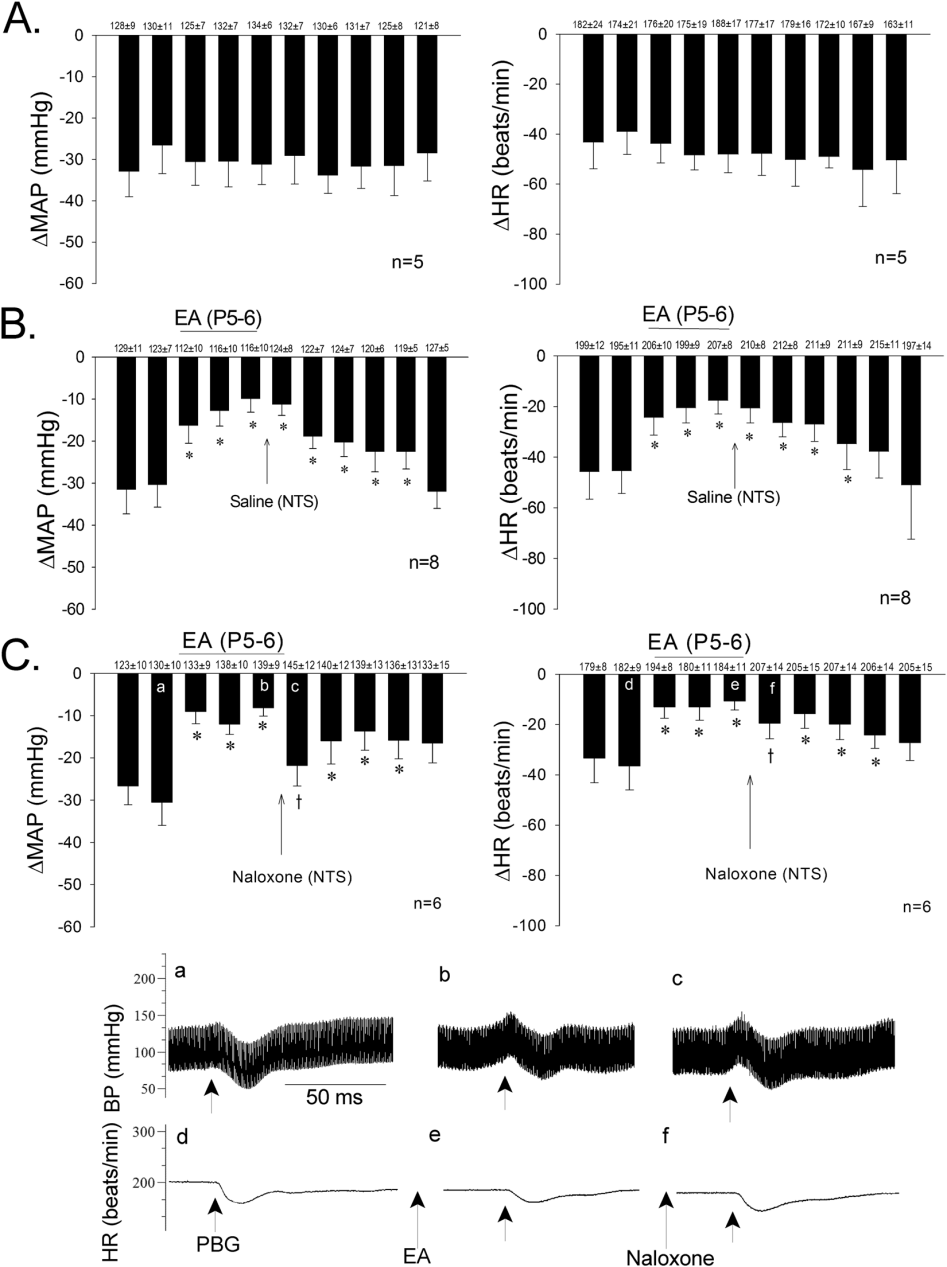
Electroacupuncture (EA) at P5–6 attenuated PBG-induced decreases in mean arterial pressure (MAP) and heart rate (HR). Bar histograms display consistent responses to repeated administration of PBG every 10 min (Panels A). EA reduced Bezold Jarisch BP responses for 50 min and bradycardia for at least 70 min. Microinjection of saline into the NTS did not influence EA induced long-lasting inhibitory cardiac reflex responses (Panels B). Opioid receptor blockade with naloxone in NTS reversed EA modulation of depressor and bradycardia responses (Panels C). *Significant difference compared with control (before EA) responses to PBG. ^†^Significant difference compared with preceding PBG reflex response during EA. Letters in (a–f) shown in the bars correspond to the original tracings showing decreases in MAP and HR (Panels c). Baseline BP and HR are shown above each bar as means ± SEM.

Repeated stimulation of cardiopulmonary serotonin receptors with PBG every 10 min without EA yielded consistent decreases in mean blood pressure (MAP) and heart rate (HR) (Fig. 1A). Naloxone or the µ-opioid receptor antagonist, CTOP, microinjected into the intermediate NTS reversed EA (P5–6) modulation of PBG-induced decreases in MAP and HR in contrast to saline control, which cause no change (Fig. 1B,C and Table 1). The inhibitory effect of EA resumed 20 min after opioid receptor blockade with either naloxone or CTOP. Conversely, in two subjects, blockade of δ-opioid receptors with ICI-174,864 did not reverse the effects of EA (−11.5 ± 5.5 vs. −9.5 ± 4.5 mmHg and −14.5 ± 2.5 vs. −12.5 ± 3.5 beats/min). In the absence of EA, blockade of opioid receptors with naloxone did not influence the Bezold Jarisch evoked MAP or HR responses (−25.5 ± 6.6 vs. −25.5 ± 7.8 mmHg; −21.5 ± 5.9 vs. −20.7 ± 4.5 beats/min) in four other animals. Microinjection of naloxone, CTOP or ICI-174,864 did not influence baseline BP or HR.

**Table 1:** Role of NTS μ-receptor activation during P5–6 EA modulation decreased mean arterial pressure (MAP) and heart rate (HR) reflex responses.

		Before EA	30 min into EA	10 min after microinjection	20 min after microinjection	60 min after EA
ΔMAP (mmHg)	Saline (4)	−30 ± 5	−14 ± 5*	−14 ± 4*	−13 ± 1*	−30 ± 4
	CTOP (4)	−29 ± 5	−16 ± 5*	−26 ± 5^†^	−19 ± 1*	−31 ± 5
ΔHR (beats/min)	Saline (4)	−28 ± 3	−14 ± 5*	−15 ± 3*	−14 ± 2*	−25 ± 4
	CTOP (4)	−21 ± 2	−13 ± 2*	−25 ± 5^†^	−15 ± 6*	−20 ± 2

*Indicates significant difference comparing PBG responses before EA with responses during and after EA.

^†^Indicate significant difference in PBG responses comparing pre with post CTOP microinjection during the effects of EA.

### Effects of point specific application of EA on PBG-evoked cardiovascular responses

**Figure 2:**
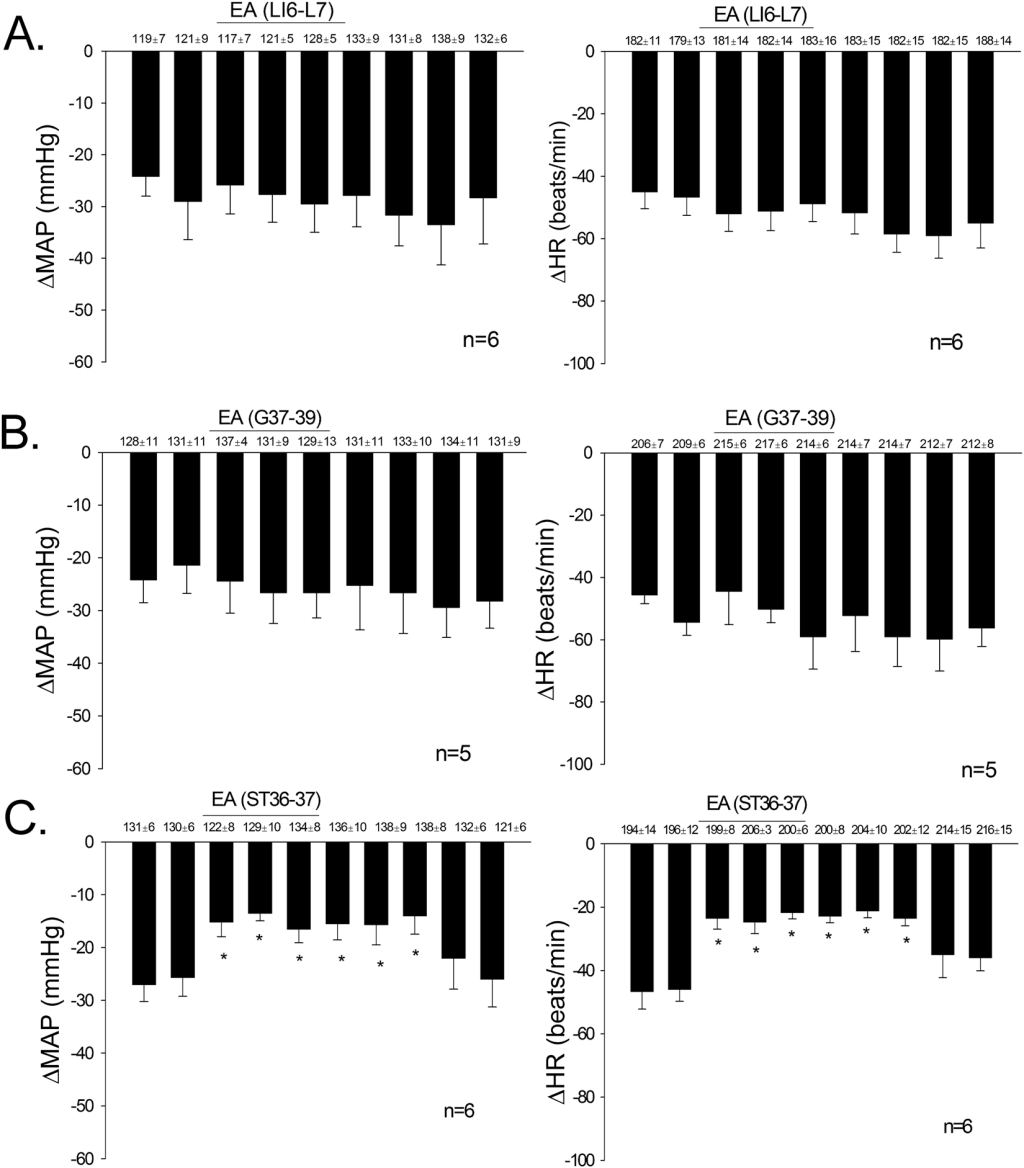
PBG-induced inhibitory cardiovascular reflex responses were examined with EA stimulation at specific points LI6–7, G37–39 or ST36–37. EA stimulation for 30 min at ST36–37 acupoints reduced the Bezold Jarisch reflex responses (Panel C) in contrast to LI6–7 (Panel A) and G37–39 (Panel B). Baseline BP and HR are shown above each bar as means ± SEM. *Significant difference compared with control responses prior to onset of EA.

The Bezold Jarisch responses were reduced following 30 min EA at P5–6 acupoints for 70 to 80 min (Fig. 1B). Stimulation at ST36–37 also modulated neurogenic inhibition of MAP and HR responses while EA at LI6–7 or G37–39 did not (Fig. 2). ST36–37 EA-inhibition on vasodepression and bradycardia lasted about 60 min. The baseline BPs and HRs before each Bezold Jarisch reflex response were not significantly different throughout the experimental protocol. EA likewise did not alter baseline BP and HR. As noted in previous studies^1,4^ gallamine triethiodide (4 mg/kg/ml), used to prevent muscle movement during application of EA, did not influence the responses to EA.

### Neuronal activity in NTS

**Figure 3:**
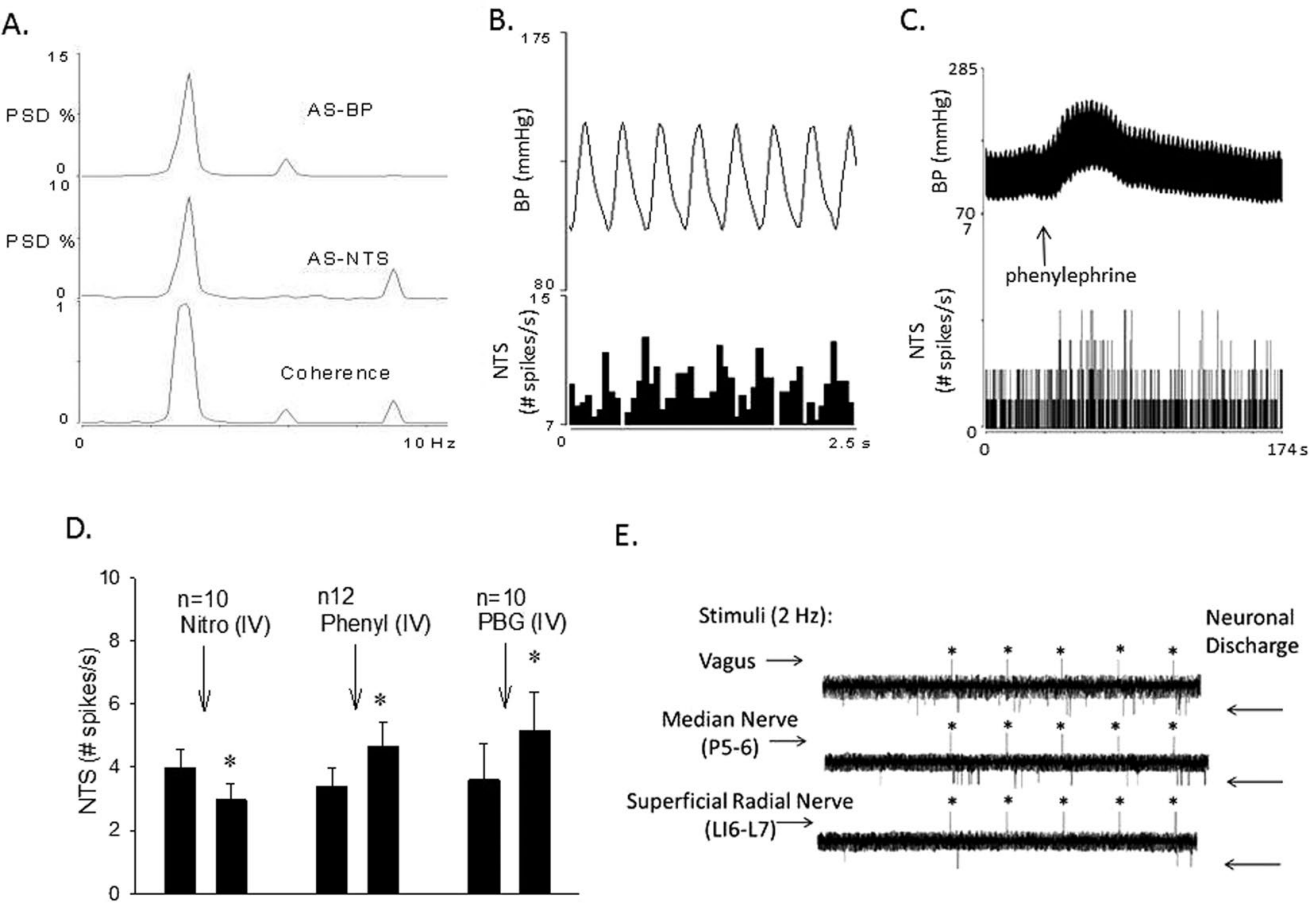
Methods used to characterize NTS neurons are displayed in Panels A-E. Panels A and B respectively show frequency (coherence at frequency of 2.5 Hz) and time domain analyses demonstrating cardiac rhythmicity. Autospectra (AS) of BP and NTS activity and corresponding coherence function of 0.97. Neuronal activity of the NTS neuron is increased with intravenous phenylephrine (Panel C). Panel D displays the group data of NTS neuronal activity in response to administration of nitroprusside-evoked decrease in BP, phenylephrine-induced increased in BP, and PBG-induced decrease in BP. *Indicates significant difference in activity after administration of nitroprusside, phenylephrine or phenylbiguanide. NTS neurons also receive input from vagus and median nerves and to a lesser degree from cutaneous nerves like the superficial radial nerve underlying LI6-L7 acupoints (Panel E). Stimulation artifact is indicated with*. Neuronal discharge is displayed before and during stimulation of vagus, median or superficial radial nerve. PSD, power spectral density.

NTS neurons included in this study were selected for a number of characteristics including cardiac rhythmicity, baroreceptor and vagal input and NTS evoked activity during stimulation of the P5–6 acupoints. Basal discharge activity of NTS neurons was 3.7 ± 0.8 spikes/sec. Thus, recordings in twenty animals, yielded forty-one cells that displayed cardiac rhythmicity (Fig. 3A and B), while a subset of 22 neurons also received baroreceptor afferent input (Fig. 3C,D) as well as convergent input from the vagus and median nerve afferents underlying P5–6. Ten neurons tested in this subset of 22 cells were activated by intravenous PBG (Fig. 3D).

### NTS opioid system in EA modulation of vagally evoked neuronal responses

**Figure 4:**
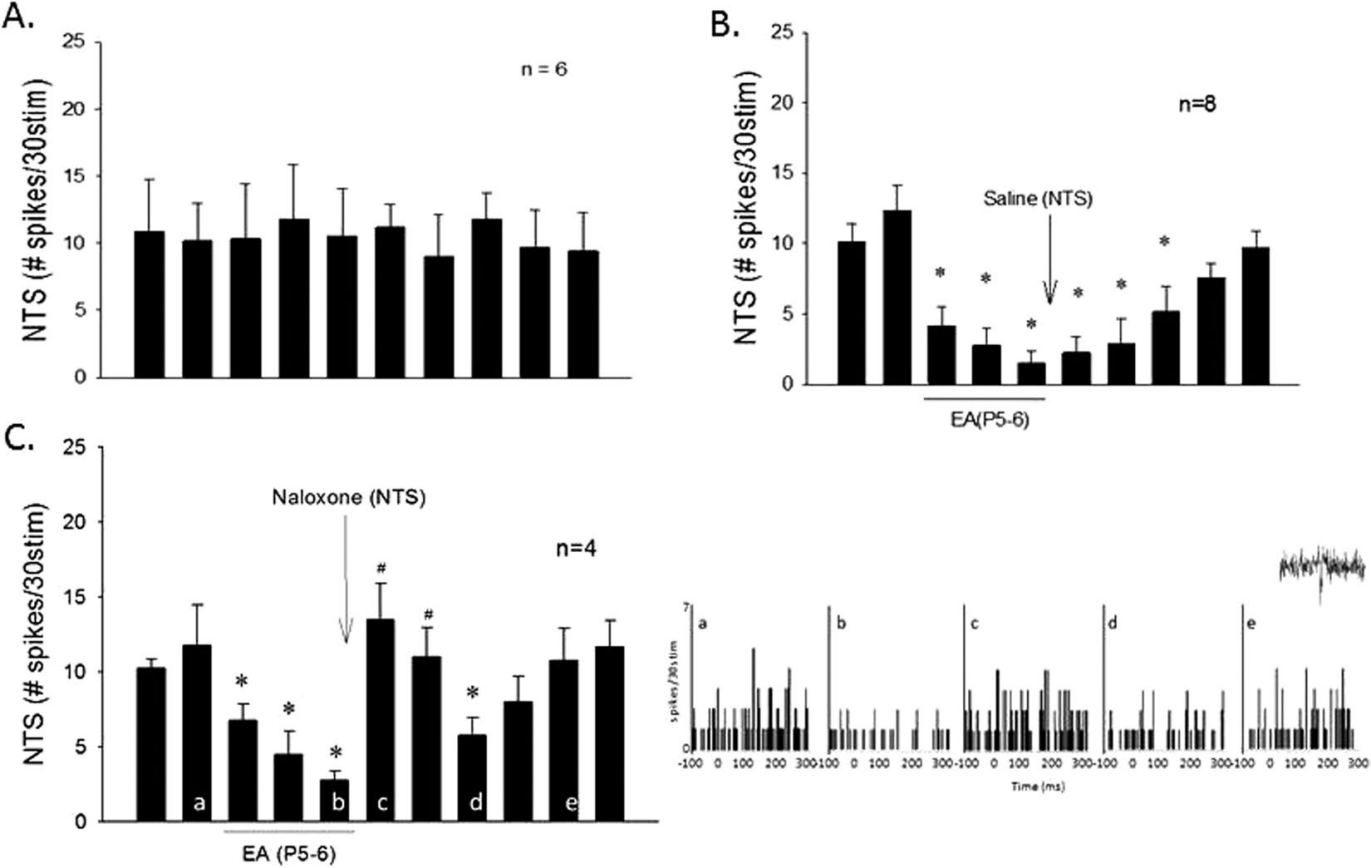
Bar histograms display vagus nerve-induced NTS neuronal activity without EA (Panel A) and with EA stimulation at P5–6 (Panels B and C). Iontophoresis of saline into the NTS did not influence the modulatory effect of EA (Panel B). *Indicates significant difference compared to activity prior to onset of EA (Panel B). Blockade of opioid receptor with iontophoresis of naloxone in the NTS reversed the EA effect (c) compared to vagal evoked activity prior to the blockade (b) for over 20 min (Panel C). Letters in a–e shown in the bars correspond with peristimulus histograms displayed to the right. *Indicates significant difference compared to control activity (a), while ^#^shows significant difference compared to preceding (b) EA response. An example of recorded spike activity is shown above e.

In the absence of EA, neurons examined during repeated stimulation of vagal afferents demonstrated consistent evoked discharge (Fig. 4A). EA applied at P5–6 (Fig. 4B) decreased the vagally evoked NTS activity during and after acupuncture stimulation for at least 60 min. Iontophoresis of naloxone into the NTS reversed EA-induced inhibition [Fig. 4C and peristimulus histograms (b-c)] for at least 20 min. Following antagonism by opioid receptor blockade, the inhibitory action of EA resumed and lasted for at least another 10 min [Fig. 4C and peristimulus histogram (d)]. Naloxone did not influence vagally evoked NTS activity (from 11.6 ± 1.7 to 11.2 ± 1.7 spikes/30 stim).

### Differential point specific effects on evoked NTS neuronal activity and point specific EA’s actions

**Figure 5:**
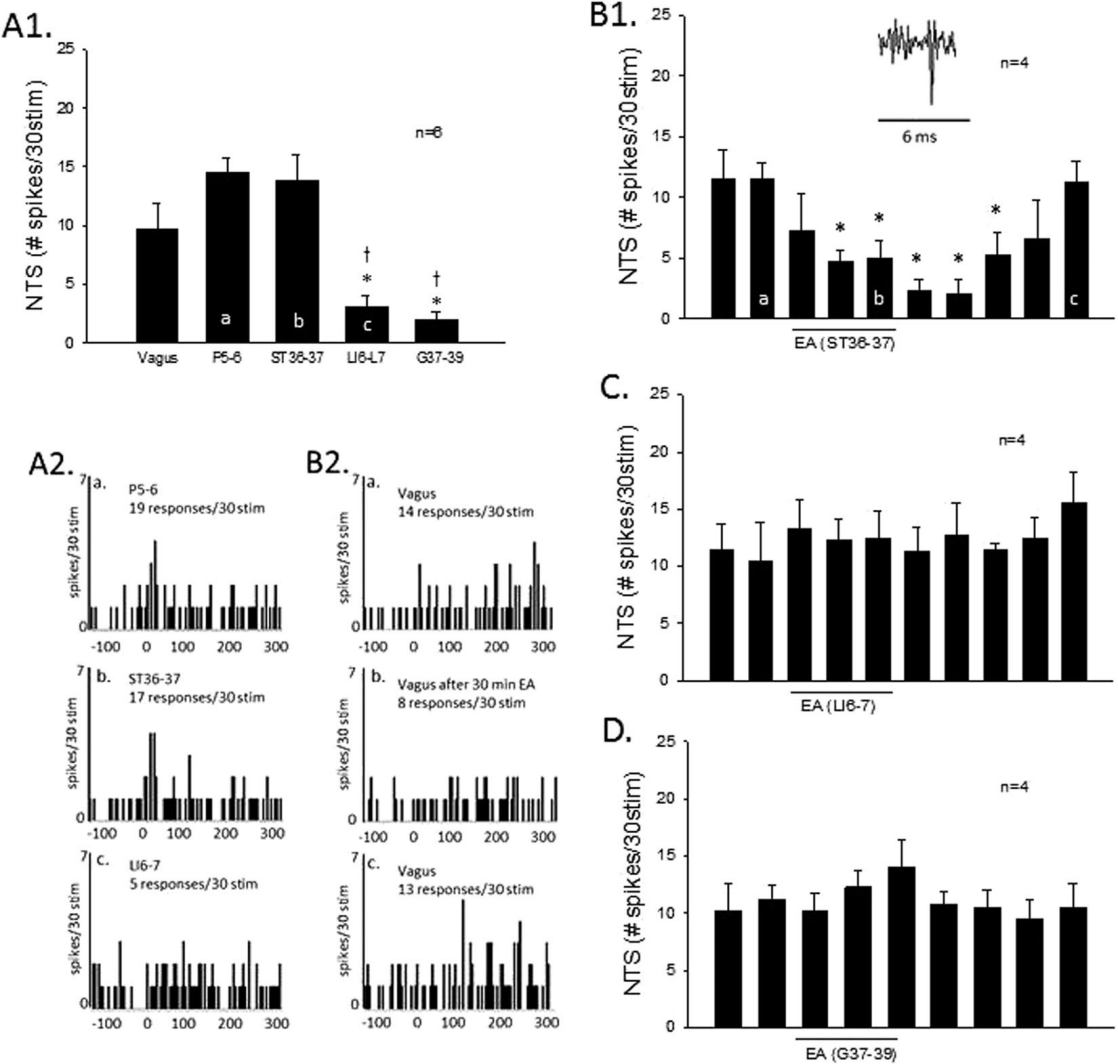
Bar graphs display inhibition of vagally evoked activity of cardiovascular NTS neurons by EA at acupoints ST36–37, LI6–7 and G37–39. Panel A1 shows group data of NTS neuronal activity evoked by brief stimulation of vagus and acupoints at P5–6, ST36–37, LI6–7 and G37–39. Examples of NTS evoked activity by brief stimulation at P5–6 (a), ST36–37 (b) and LI6–7 (c) are shown in Panel A2. Peristimulus time histograms a, b, c correspond to letters in bars shown in Panel A1. Thirty-min EA application at ST36–37 inhibited vagally evoked NTS activity shown in Panel B1. Inset displays a vagally evoked NTS action potential. Panel B2 displays peristimulus histograms of NTS neuronal activity with letters a, b and c corresponding to the bar graph in Panel B1. In contrast to ST36–37 (Panel B1), EA at LI6–7 or G37–39 did not inhibit the vagally evoked NTS discharge (Panels C and D). (^†^), *In Panel A1 indicate significant difference comparing LI6–7 and G37–39 evoked activity to vagally evoked discharge (^†^) and to P5–6 and ST36–37 induced activity (*). *In Panel B1 indicates significant difference compared to activity before onset of EA.

Cardiovascular NTS neurons displayed differential evoked responses during brief stimulation at acupoints P5–6, ST36–37, LI6–7 or G37–39. Brief stimulation of the vagus nerve and acupoints at P5–6 and ST36–37 (overlying median and deep peroneal nerves) evoked significant activity in the same NTS neurons, in contrast to the small evoked responses during  stimulation at LI6–7 and G37–39 (superficial radial and peroneal nerves) (Fig. 5A). In addition to P5–6 EA-inhibition, we also observed that the vagally evoked NTS activity was reduced by 30 min of EA at ST36–37 (Fig. 5B) but not by stimulating LI6–7 or G37–39 (Fig. 5C,D).

### Histology

**Figure 6:**
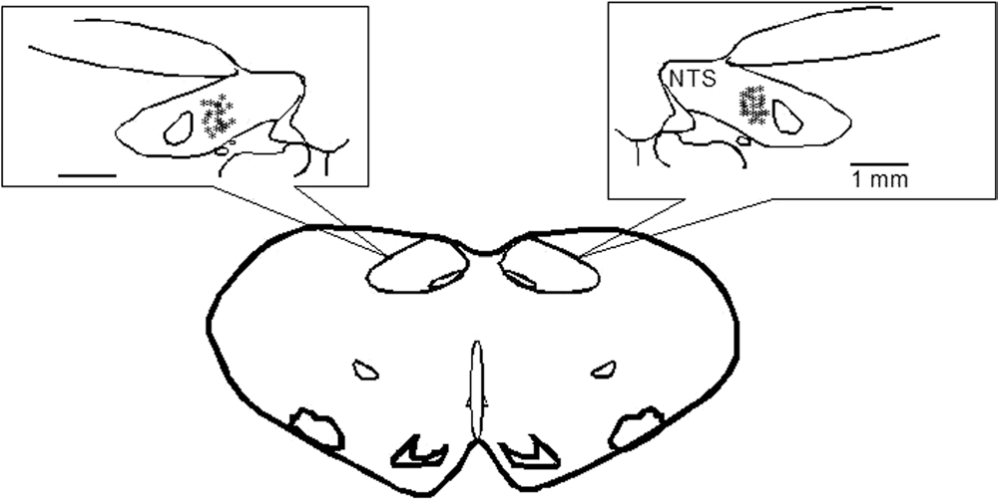
Composite map displays sites of microinjections, iontophoresis and extracellular recordings in intermediate NTS of cats. For ease of representation, all recording sites are displayed on the right and microinjections on the left. (*), sites located within NTS. O, control site outside intermediate NTS. Coronal section is 0 to 0.6 mm rostral to obex.

Microinjection and recording sites in the NTS were confirmed histologically, as shown in Berman’s atlas^32^. Sites displayed on the composite map (Fig. 6) were 1.6 to 2.2 mm lateral to the midline, 0.7 to 1.4 mm depth from the dorsal surface and at 0.6 to 0 mm rostral to the obex. Each site was located by microinjection tracks or dye spots.

## Discussion

The present study documents the role of opioids in the NTS in EA-inhibition of the vagally evoked Bezold Jarisch response, a surrogate of neurogenic syncope. Blockade of opioid receptors, particularly μ-opioid receptors, but not δ-receptors in the NTS, reversed P5–6 EA modulation of the reflex induced vasodepression and bradycardia. The current findings also showed that EA at certain  acupoints, including P5–6 or ST36–37, reduced the PBG-induced hemodynamic responses for at least 60 min. Conversely, EA-inhibition was not effective following stimulation at either LI6–7 or G37–39 acupoints. We also observed that the activity of cardiovascular NTS neurons was influenced by PBG and received input from the vagus nerve and afferent neural pathways underlying the acupoints. The differentially evoked activities and EA-inhibition of NTS neurons by electrical stimulation at select acupoints were congruent with the EA actions on hemodynamic responses. We thus found that the NTS cells were activated significantly by brief stimulation at P5–6 or ST36–37, unlike stimulation at LI6–7 or G37–39. In agreement with differential point specific inputs, vagal-evoked NTS activity was reduced by EA applied at ST36–37 or P5–6 but not at LI6–7 or G37–39. Modulation by EA of NTS neuronal activity was reversed with local opioid receptor blockade. Thus, activation of μ-opioid receptors and EA at P5–6 or ST36–37 contribute to EA inhibition of vagally evoked NTS activity, vasodepression and bradycardia responses.

Neuroanatomical and electrophysiological studies have shown that NTS neurons process visceral sensory vagal input that originate from baroreceptors and the cardiopulmonary region, among other sources^1,29,33^. Specifically, injection of PBG into the right atrium activates cardiopulmonary 5-hydroxytryptamine 3 (5-HT3) receptors located on endings of vagal C-fiber afferents^34–36^, cardiovascular vagally evoked NTS neurons^1,37^ and glutamatergic NTS receptors^38^. PBG also leads to sympathoinhibition (vasodepression and bradycardia) through stimulation of splanchnic afferents^1,4,38^. The current study shows that opioid receptors in the NTS are not involved in neurogenic-evoked reflex responses but play an important role in EA-inhibition of PBG-induced vasodepression and bradycardia. Thus, NTS neurons integrate prolonged EA-activated somatosensory afferent input through a neuromodulatory opioid mechanism but do not normally use this system to process cardiopulmonary vagally evoked activity in the absence of somatic stimulation.

µ-opioid receptors are distributed throughout the CNS including the NTS^39^. Immunocytochemical observation has shown that β-endorphinergic cell bodies are located in the NTS^40^. Another labeling study reported that NTS μ-opioid receptors are located in close proximity to enkephalinergic fibers^39^ suggesting that enkephalins potentially are an endogenous ligand for these opioid receptors. Pharmacological stimulation of μ-opioid receptors reduces reflex responses during carotid occlusion and baroreceptor activation^30,41^. The current study shows that blockade of µ- but likely not δ-opioid receptors reverses EA inhibition of PBG-induced inhibitory cardiovascular reflex responses in the NTS. These observations indicate that endorphins preferentially through μ-opioid receptors and enkephalins through δ-opioid receptors participate in these EA actions. Thus, stimulation of µ-opioid receptors by endorphins, and less likely enkephalins, in the NTS facilitates modulation by EA of reflex vasodepression and bradycardia.

Our current and previous findings demonstrate that EA in a point specific manner inhibits both parasympathetically induced as well as sympathetically evoked activity in the NTS and rVLM, respectively^14^. These findings confirm that EA stimulation of specific acupoints, including P5–6 and ST36–37 overlying deep nerves (median and deep peroneal nerve), but not LI6-L7 and G37–39 acupoints overlying more superficial or cutaneous nerves, modulate both hypotensive and hypertensive responses. Several other brain imaging studies evaluating acupuncture responses in other diseases indicate point-specific neuronal responses to acupuncture stimulation^42–44^. For instance, Zhang *et al*. using brain imaging demonstrated that specific acupoints used for different problems affecting adrenocortical glands, muscle and tendons activate select regions identified with brain MRI^43^. Another fMRI imaging study reported that stimulation of P6 or an inactive point near P6 activates a number of similar regions in the brain, while stimulation of P6 alone activates other brain areas^44^ that are unique to acupuncture at P6. We have approached this question by recording single neuron activity in cardiovascular regions during point specific stimulation^14,19^. We found that, in contrast to superficial nerves, stimulation of the deep nerves underlying acupoints P5–6 and ST36–37 provides significant input to cardiovascular NTS cells. The fact that deep afferent pathways, but not cutaneous fibers, deliver greater input in the NTS provides an anatomical explanation for greater modulation by EA of reflex vasodepression and bradycardia by some acupoints and not others, hence suggesting a mechanism for acupuncture’s point specific actions. This is consistent to what we have observed during acupoint stimulation in other nuclei like the rVLM that regulate sympathetic outflow^14^.

Although opioid receptor blockade reverses EA-inhibition of both vasodepression and bradycardia during EA, GABA_A_ receptors in the intermediate NTS contribute to EA-related modulation of only the bradycardia but not the PBG evoked vasodepressor response^1^. Machado *et al*.^45^ reported that in the lateral commissural region of the NTS, stimulation of GABA_A_ receptors inhibits cardiac chronotropic responses while GABA_B_ receptors influence BP responses to chemoreflex activation. Furthermore, EA modulates the PBG induced bradycardia mediated through descending parasympathetic NTS-NAmb projections^1,4^. The Bezold Jarisch reflex also involves an NTS-cVLM-rVLM pathway^28^. Modulation by EA of cardiopulmonary and gastric afferent evoked vasodepression respectively in the NTS^1^ and cVLM and rVLM^9^ suggests that EA-inhibition of hypotension likely involves a number of descending pathways.

### Perspectives and Significance

Activation of cardiopulmonary vagal afferents with PBG to lower BP and HR shares many features of vasovagal syncope. This form of neurogenic syncope is clinically the most common cause of transient unconsciousness^46^ and is thought, at least in part, to be mediated by activation of a cardiopulmonary reflex^3^. Our previous studies have shown that acupuncture decreases cardiopulmonary inhibitory responses and bradycardia through a GABA_A_ mechanism in the NAmb and NTS^1,4^. The current study underscores the importance of opioids in the NTS and the potential of acupuncture at specific acupoints to minimize the vasodepression and bradycardia responses induced by vagal activation. Parasympathetic activation underlies many hypotensive responses, for instance those occurring during alveolar hemorrhage, hypercapnia and vasovagal syncope^25,26,47^. Excitation of vagal pulmonary C-fibers in response to hemorrhage, chemical stimuli such as CO_2,_ capsaicin and PBG profoundly decreases BP and HR that potentially can be modulated by EA^4,9,25,26,47,48^. Of note, EA-inhibition does not influence baseline BP and HR^16,49–51^, suggesting that EA does not influence tonic autonomic output or hemodynamic stability. This latter observation is consistent with the low incidence of side effects during EA application^14,49^. Thus, EA, without altering baseline BP, may serve as therapeutic modality for patients with recurrent cardiovascular depression. Additional clinical studies need to be conducted to verify acupuncture’s role in syncope or other conditions associated with bradycardia and hypotension, for instance during hemorrhage and hypercapnia.

In conclusion, 30 min of EA in a point specific manner decreases vagally evoked cardiovascular NTS neuronal activity, vasodepression and bradycardia through a µ-opioid mechanism.

## Materials and Methods

### Surgical Procedures

The animal use and care committee at the University of California, Irvine, approved all surgical and experimental protocols in this study. All procedures were carried out in accordance with the US Society for Neuroscience and the National Institutes of Health guidelines. Steps were taken to minimize discomfort and suffering of the animals throughout the study. Both male and female cats were preanesthetized with ketamine (40 mg/kg, sc). A venous injection of α-chloralose (50 mg/kg) was administered to induce anesthesia. A femoral vein then was cannulated for further administration of drugs and fluids. Intravenous (iv) supplemental α-chloralose (5–10 mg/kg,) was given to maintain adequate depth of anesthesia if the animals exhibited a corneal reflex, withdrew a limb in response to a noxious stimulus during the experiment or displayed an unstable respiratory pattern of BP. A femoral artery was cannulated for measurement of arterial BP (Statham P 23 ID, Oxnard, CA, USA). HR was derived from the arterial BP pulse using a biotech Gould Instrument (Cleveland, OH, USA). BP and HR were recorded and analyzed offline with a computer and CED Spike 2 Windows software. Intubation of the trachea facilitated artificial respiration with room air enriched with oxygen (Harvard pump, model 662, Ealing, South Natick, MA, USA). Throughout the experiment arterial blood gases were examined (Radiometer, Model ABL-3, Westlake, OH, USA) and maintained within the normal physiological range (PO_2_, 100–150 mmHg; PCO_2_, 28–35 mmHg; pH 7.35–7.45) by iv administration of 8% sodium bicarbonate or by adjusting the ventilator. A heating pad and an external heat lamp were used to maintain body temperature between 36 and 38 °C.

The other femoral vein was cannulated to position the tip of a cannula close to the right ventricle to administer PBG. A lateral thoracotomy on the right was performed between the fourth and fifth ribs. The ribs were cut to access the cardiac branch of the vagus nerve. A bipolar flexible platinum electrode was placed around the vagus nerve and held in place with dental impression material (polyvinyldimethylsiloxone, Pentron, Wallington, CT). The stimulating electrode was connected to an isolation unit and a stimulator (Grass, model S88). To confirm isolation of the cardiac branch, the nerve was stimulated transiently (0.4 mA, 10 Hz and 0.5 ms) to elicit a decrease in HR^4^. In other animals, the cervical vagus was isolated and stimulated (0.7 to 1 mA, 10 Hz and 0.5 ms) with a bipolar electrode held in place with the dental impression material to elicit decreases in HR. The thoracic wall and lateral cervical incisions were closed to prevent desiccation and heat loss. After the animal was stabilized with a Kopf stereotaxic head frame, a craniotomy was performed and the dorsal surface of the medulla was exposed to access the NTS.

A microinjection probe consisting of a stainless steel guide tube with an outer diameter of 0.75 mm and an injection cannula with an inner diameter of 0.4 mm was inserted into the NTS to examine the inhibitory cardiovascular responses. The probe was inserted with visual approximation into the intermediate NTS, a site sensitive to PBG, at a location 2 mm lateral to the midline at the level of the obex, according to coordinates taken from Berman’s atlas^1,32^. Unilateral insertion of the microinjection probe allowed maintenance of a more optimal physiological condition compared to bilateral electrode insertion. A one- or three-barrel glass pipette electrode was used to evaluate neuronal activity or evaluate neuronal activity before and after iontophoresis of the opioid receptor antagonist or saline. One barrel of the glass pipette was filled either with naloxone or saline. The second barrel contained a platinum recording electrode in 0.5 M sodium acetate and 2% Chicago sky blue (Sigma Chemical, St. Louis, MO), while the third barrel was filled with 4 M NaCl to balance the current. A one or three-barrel glass pipette or microinjection probe was positioned perpendicularly to the dorsal surface of the medulla using visual approximation, 0 to 0.5 mm rostral to the obex and advanced ventrally approximately 0.8 mm to reach the NTS. At end of experiment, the recording and microinjection sites of drugs into the NTS were marked with Chicago blue dye for later histological confirmation.

### Methods of Blockade

The roles of opioid receptors in the NTS during EA were evaluated by unilateral microinjection of the non-specific antagonist naloxone (100 nM, 50 nl, Sigma Aldrich, St. Louise, MO)^16^, CTOP (μ-opioid antagonist, 10–20 nM, 50 nl)^52^ or ICI-174,864 (δ-opioid receptor antagonist, 30–100 nM, 50 nl)^53^ 5 min after terminating EA stimulation, at a time when EA-inhibition of the Bezold Jarisch reflex bradycardia and vasodepression were still present (as shown in control studies). Several of our previous studies^16,52^ have demonstrated significant blockade with unilateral microinjection of naloxone, CTOP or ICI174,864 to demonstrate the roles of opioids in various nuclei^4,16,54^. Unilateral microinjection allows maintenance of optimal physiological state in studies of EA-cardiovascular responses^4,52^. Iontophoresis (Neuro Phore BH-2 system, Medical System, Greenvale, NY) of saline as a control or naloxone into the NTS was applied for approximately 2 min at the end of acupuncture stimulation. The antagonist also was iontophoresed during repeated stimulation of the cardiac vagus nerve in the absence of EA. A current of 120–130 nA was used for iontophoresis. A negative current of 5–10 nA was used to prevent leakage.

### Stimulating Methods

Stimulation of cardiopulmonary serotonin receptors with PBG (40 µg/ml/kg, iv) or the cervical vagus and vagal cardiac branch was repeated every 10 min. The vagus nerves were stimulated to provide afferent input to the NTS^1,4^. Acupuncture needles were inserted bilaterally at the following acupoints: Neiguan-Jianshi (P5–6, placed to a depth of about 4 mm above the wrist overlying the median nerves), Zusanli-Shangjuxu (ST36–37, placed at a depth of about 5 mm on the anterolateral side of the hind-limb near the anterior crest of the tibia below the knee, under the tibialis anterior muscle that overlies the deep peroneal nerves), Pianli-Wenliu (LI6–7, inserted at a 20° angle on the radial side of the dorsal surface on the lower one-third of the forelimb between the abductor and extensor pollicus longus muscles that overlie the superficial radial nerves) or Guanming-Xuanzhong (G37–39, placed at a 20° angle to the lateral surface of the leg above the ankle at the lower one third of the hindlimb overlying superficial peroneal nerves). The needles were connected to an isolation unit and stimulator (Grass, model S88) that delivered bipolar stimuli at 2 Hz, 0.5 ms and 2–4 mA^16^. The somatic and cutaneous nerves beneath P5–6, ST36–37, LI6–7 or G37–39 acupoints were stimulated bilaterally for 30 min to simulate clinical use of EA^51,52^. Gallamine triethiodide (4 mg/kg) was administered intravenously before application of EA or recording neuronal activity to avoid muscle movement during stimulation of somatic nerves.

### Extracellular NTS Recordings

Single-unit activity of NTS neurons was recorded with a platinum electrode inserted in a glass barrel pipette filled with 0.5 M sodium acetate containing 2% Chicago sky blue. The electrode was positioned using visual approximation into the NTS 0.1 mm rostral to the obex, 2.0 mm lateral to the midline and 0.8 mm depth from the surface of the medulla. Action potentials were amplified with a preamplifier (Neuroprobe Amplifier Model 1600, A-M Systems, Inc.) attached to a Nerve Traffic Analysis System 662C-3 (Bioengineering, College of Medicine, University of Iowa), then filtered (0.3–10 KHz) and monitored with an oscilloscope (Tektronix 2201). Action potentials, blood pressures and heart rates were digitized and analyzed online with a computer and a four-channel data acquisition system program (SHMU; Shanghai Medical College of Fudan University, China) that uses wave shape recognition algorithms to allow detection of similar wave shapes, heights and latencies of response^18,55^. Peristimulus time histograms were constructed for each neuron to assess evoked responses to stimulation of the vagus or acupoints overlying the median, deep peroneal, superficial radial and peroneal nerves. The relationship between NTS neuronal activity and BP was assessed by both time and frequency domain analyses using arterial pulse triggered averaging and coherence analysis^14–16,18^. Examination of the responses to baroreceptor afferent input with either nitroprusside (50 μg/kg) or phenylephrine (2.5 μg/kg) provided additional characterization of NTS neurons. Cardiovascular NTS neurons that received convergence from vagal and baroreceptor afferents, displayed cardiac rhythmicity and increased activity with brief (30 to 60 sec) stimulation at the four sets of acupoints were subjected to EA stimulation. The EA effects on the vagal-evoked NTS neuronal activity were evaluated after 30 min of stimulation at acupoints P5–6, ST36–37, LI6–7 or G37–39.

## Experimental Protocols

### PBG evoked reflexes

BP and HR were lowered repeatedly every 10 min by stimulating cardiopulmonary afferents with intravenous injections of PBG. Maximal decreases in mean MAP and HR were recorded as the change in MAP and HR before application of PBG to the lowest MAP and HR during the reflex responses.

### NTS opioid system in EA-modulation of cardiopulmonary reflex responses

Consistency of vasodepression and bradycardia responses to PBG was evaluated in a group of five animals (Fig. 1a). EA-inhibition (P5–6) of the decreases in MAP and HR was examined in eight other animals. As a control for the receptor blockade studies, saline was microinjected into the NTS 5 min following termination of EA in six of the eight subjects tested for P5–6 EA-inhibition (Fig. 1b). Opioid receptors in the NTS were blocked to evaluate their role in mediating the actions of EA on hypotension and bradycardia. In this regard, either the opioid receptor antagonist naloxone or CTOP was microinjected 5 min after termination of EA into the NTS in another 10 animals (Fig. 1c and Table 1). We also evaluated NTS δ-opioid receptor blockade using ICI-174,864, 5 min following EA application in two additional subjects. To confirm the role of opioids in EA-inhibition, the effect of naloxone on the inhibitory cardiopulmonary responses in the absence of EA was examined in four other animals.

### Point specific EA-inhibition of vasodepression and bradycardia

Point specific EA-modulation of cardiopulmonary reflex responses was determined by stimulating three additional sets of acupoints, including ST36–37, LI6–7 or G37–39. In this protocol, after obtaining two repeatable PBG responses, eight additional reflex responses were evaluated during and after 30 min of EA at one set of acupoints in 17 additional animals (Fig. 2). BP and HR decreases were evaluated every 10 min to determine the effectiveness of EA-inhibition.

### Electrophysiological studies in NTS

We identified NTS neurons (Fig. 3) that received baroreceptor input by iv administration of nitroprusside or phenylephrine. Ten NTS neurons also were evaluated for responsiveness to intravenous PBG. Each neuron demonstrated cardiac rhythmicity measured by assessing time and frequency domain relationships between arterial BP and cellular activity (pulse triggered activity and coherence, respectively). Selected cardiovascular NTS neurons then were studied with respect to the role of opioid receptors in EA-inhibition of vagally evoked responses, point specific convergence and actions of EA.

### NTS opioid system in EA-modulation of neuronal responses

Selected neurons in the NTS were activated every 10 min by stimulation of the cardiac or cervical vagus nerves. Peristimulus histograms constructed with histogram bars represented vagally evoked activity over and above the basal neuronal discharge rate. Consistency of evoked neuronal responses with 10 repeated vagal nerve stimuli were examined in six cardiovascular NTS neurons (Fig. 4A).

The influence of opioid receptor blockade was assessed by iontophoresis of naloxone 5 min following termination of EA at P5–6 in four NTS cells (Fig. 4C). In the absence of EA, the effect of opioid receptor blockade in the NTS also was evaluated with repeated vagal afferent evoked activity in these four cells. The two studies of repeated vagal stimulation randomly ordered with and without EA at P5–6 were separated by 30 min. Saline control iontophoresis after EA stimulation at P5–6 was evaluated in four other cells (Fig. 4B). A total of 14 NTS neurons were examined to identify the role of opioid receptors in EA-inhibition of cardiopulmonary vagal responses.

### Point specific convergence in NTS

Cardiovascular NTS neurons were evaluated during stimulation at one of the four sets of acupoints overlying the median nerve, deep peroneal nerve, superficial radial nerve or superficial peroneal nerve. Thus, NTS neurons were stimulated briefly with acupuncture needles placed at the P5–6, ST36–37, LI6–7 or G37–39 acupoints or vagus nerve to determine stimulation evoked activity in the NTS. Convergence of somatic nerves in six of eight NTS neurons (Fig. 5A) examined for opioid receptor blockade was evaluated a half hour either before or after the cell was exposed to EA-inhibition at P5–6.

### Point specific EA-inhibition on NTS responses

In eight other NTS cells, in random order and 30 min apart, the effects of 30 min of EA were examined by stimulating: (1) P5–6 or LI6–7, (2) P5–6 or G37–39, (3) G37–39 or ST36–37, (4) LI6–7 or ST36–37 with PBG-induced responses. Thus, in this protocol 14 cells were examined for consistency of vagally evoked responses (n = 6) and EA stimulation at acupoints P5–6 (n = 4, Fig. 4B), ST36–37 (n = 4, Fig. 5B), LI6–7 (n = 4, Fig. 5C) and G37–39 (n = 4, Fig. 5D).

### Histology

At the end of each experiment, animals were euthanized under deep α-chloralose anesthesia followed by iv injection of saturated KCl. Recording and/or microinjection sites were marked by iontophoresis and/or microinjection of 2% Chicago blue dye. Thereafter, the brain was removed and fixed in 10% paraformaldehyde for at least 2 days. Brain stems were sliced with a microtome cryostat into 60-µm coronal sections. Recording and microinjection sites were reconstructed from the dye spots with the aid of a microscope (Nikon) and software (Corel presentation). The sites were plotted on coronal sections separated by 0.6 mm rostral to the obex^32^.

### Statistical Analyses

Data are presented as means ± SEMs. Evoked activity was measured as the increase in number of spikes above baseline neuronal discharge. Changes in MAP and HR are presented as bar histograms. The increase in cellular activity and decreases in MAP and HR before and after delivery of opioid antagonists or saline were compared by a one-way ANOVA followed post hoc with the Student-Newman Keuls test. Point specific responses were compared using a one-way repeated measures of ANOVA followed post hoc with the Student-Newman Keuls test. Data were plotted and analyzed with the Kolmogorov-Smirnov test for normal data distribution and normalized when necessary with Sigma plot (Jandel Scientific). Statistical analyses were performed with Sigma Stat (Jandel Scientific). The 0.05 probability level was used to detect significant differences.

To determine cardiac rhythmicity, we evaluated time and frequency relationships between NTS neuronal activity and arterial BP using pulse-triggered averaging. Pulse triggered averaging used a threshold set at the systolic phase of the arterial pulse while spike height discrimination and waveform recognition were used to sort action potentials during a 300 sec evaluation period. Averages of the arterial pulse and histograms of NTS neuronal activity were constructed with time domain analysis as described in our previous studies^1,21,56^. Frequency domain analysis involved assessment of the coherence between NTS activity and arterial BP using a Fast Fourier Transform (FFT) algorithm. We recorded data using a sampling rate of 10,000 Hz. Reconstructed data utilized every tenth sample, including assessment of the mean and peak amplitudes and the maximum and minimum slopes of the original spike to preserve the action potentials. The spikes were sorted and identified with a window discriminator to construct histograms prior to coherence analysis. The number of data sections (15–20 each lasting for 12.8 s) was chosen to determine the average histogram. Autospectra of NTS discharge and arterial BP were generated with an FFT algorithm^57^. Thus, coherence was generated with seven overlapping windows, each with a length of 12.8 s, consisting of 256 bins, with bin widths of 50 ms. The auto-spectral analysis was adopted from Shin *et al*., 1995^58^ using contiguous segments of 256 beats with 50% overlap between the segments. The frequency resolution was 1/12 s or 0.08 Hz. Coherence (normalized cross-spectrum) provides a measure of the strength of linear correlation of NTS neuronal activity and BP at each frequency. Coherence values of ≥0.5 were chosen to reflect a statistically significant relationship between NTS spikes and arterial BP^16^.
